# Reprogramming of Plant Central Metabolism in Response to Abiotic Stresses: A Metabolomics View

**DOI:** 10.3390/ijms23105716

**Published:** 2022-05-20

**Authors:** Yuan Xu, Xinyu Fu

**Affiliations:** 1 Department of Plant Biology, Michigan State University, East Lansing, MI 48824, USA; 2 Plant Research Laboratory, Michigan State University, East Lansing, MI 48824, USA

**Keywords:** plant central metabolism, abiotic stresses, metabolomics, flooding, drought, salt, heat, freezing

## Abstract

Abiotic stresses rewire plant central metabolism to maintain metabolic and energy homeostasis. Metabolites involved in the plant central metabolic network serve as a hub for regulating carbon and energy metabolism under various stress conditions. In this review, we introduce recent metabolomics techniques used to investigate the dynamics of metabolic responses to abiotic stresses and analyze the trend of publications in this field. We provide an updated overview of the changing patterns in central metabolic pathways related to the metabolic responses to common stresses, including flooding, drought, cold, heat, and salinity. We extensively review the common and unique metabolic changes in central metabolism in response to major abiotic stresses. Finally, we discuss the challenges and some emerging insights in the future application of metabolomics to study plant responses to abiotic stresses.

## 1. Introduction

Plants are constantly exposed to a plethora of stresses under natural conditions. Stress in plants can be described as anything that can cause a change from ideal growth and developmental conditions [1]. Stresses can be classified as abiotic or biotic, where abiotic stresses are caused by nonliving factors in the surrounding environment, such as extremes in temperature, drought, flooding, and high salinity [2]. Abiotic stresses are unavoidable to plants due to their inability to move [3]. Global warming and climate change result in increases in the frequency and intensity of abiotic stresses, such as heatwaves, cold snaps, droughts, and floods [4,5,6,7]. These abiotic stresses are the primary causes for the reduction in crop yield and quality and may threaten food security [8,9]. The economic losses caused by abiotic stresses are estimated to be around USD 14–19 million yearly, worldwide [10]. Therefore, five abiotic stresses, including drought, flooding, salinity, cold, and heat, which strongly impact crop yield and the food industry are discussed in this review.

Heat stress has become a global concern that adversely affects crop yield worldwide because of global warming, with steadily increasing ambient temperatures over the past 40 years with frequently occurring heat waves [11,12]. Global warming leads to climate change and could exacerbate drought stress. Drought stress occurs due to various environments, such as temperature dynamics, light intensity, and low rainfall, and is the leading abiotic stress that hampers crop productivity and threatens food security worldwide [13]. Soil flooding is one of the most important abiotic stresses in wetland and high-rainfall areas in crops and woody tree species [14,15]. It is estimated that 10% of the global land area is affected by soil flooding or severe soil drainage constraints [16]. These climate disasters can cause harsh soil conditions, for example, high soil salinity, extreme pH, and high level of environmental pollutants, such as heavy metals, polycyclic aromatic hydrocarbons (PAHs), herbicides, and pesticides [7]. Salinity stress affects about one-third of the irrigated land on earth by uneven rainfall, coastal lands flooded with seawater, and poor quality of irrigation water as a result of groundwater depletion and degradation of high-salt rocks [17]. Cold stress impacts the reproductive development of chilling sensitive, tropical, and subtropical crops, and is judged to be the major abiotic stress for seedlings [18]. The yield reduction in food crops caused by abiotic stresses worldwide is considered a major challenge in agronomy [19]. Thus, understanding plant responses to various abiotic stress events is central in plant research.

Plant metabolism responds sensitively and dynamically to various abiotic stresses. Metabolic responses to stresses can be very rapid, making metabolic changes an important feature of plant stress responses [20]. The metabolic perturbations under abiotic stresses can be caused by inhibition of metabolic enzymes or lack of specific substrates or cofactors [21]. The plant metabolic network has to be reprogrammed under stresses so that essential metabolic homeostasis is maintained and protective metabolites are produced to enhance stress tolerance [21]. Exogenous application of metabolites, such as amino acids, sugars, and specialized metabolites (secondary metabolites), has proven to effectively increase stress tolerance in various crop plants [17,22,23,24]. Evolving metabolomics approaches have shed light on the regulation of central metabolism and specialized metabolism under abiotic stresses [1,25,26,27,28,29]. The response of genes and specialized metabolites to abiotic stresses varies among species and have been extensively reviewed recently [30,31,32,33,34,35,36,37,38]. Common stress-induced specialized metabolites include flavonoids, terpenes, phenols, and alkaloids, synthesized in certain species, organs, tissues, and cells [31,32,33]. However, a comprehensive review of plant central metabolic changes in response to different abiotic stresses is needed. Many excellent reviews summarize various metabolic responses under a specific stress, we aim to systematically examine several key abiotic stresses to reveal the reprogramming of plant central metabolism with a network-wide perspective.

Plant central metabolism functions as a hub to quickly adjust metabolic demands in response to various abiotic stresses. The reconfiguration of metabolic fluxes in central metabolism upon abiotic stresses is highly conserved in plant species [21]. It is essential to understand the regulation of central metabolites if we are to rationally modify plant systems to maximize plant resilience to various abiotic stresses. In the following sections, we briefly introduce the recent metabolomics techniques used to investigate plant metabolism. We review the key metabolic responses to the major abiotic stresses, such as flooding, drought, cold, heat, and salinity. We focus on discussing the changes in metabolites involved in central metabolic pathways, such as the Calvin–Benson cycle (CBC), photorespiration, glycolysis, the tricarboxylic acid (TCA) cycle, and the metabolism of sugars, sugar alcohols, and amino acids. Recent metabolomics studies that identify common and unique signatures of central metabolites in response to the five major abiotic stresses are discussed. This review highlights the recent advances in understanding the metabolic reprogramming in plant responses to abiotic stresses with an emphasis on central metabolism.

## 2. Using Metabolomics as a Tool to Study Plant Abiotic Stress Responses

**Figure 1:**
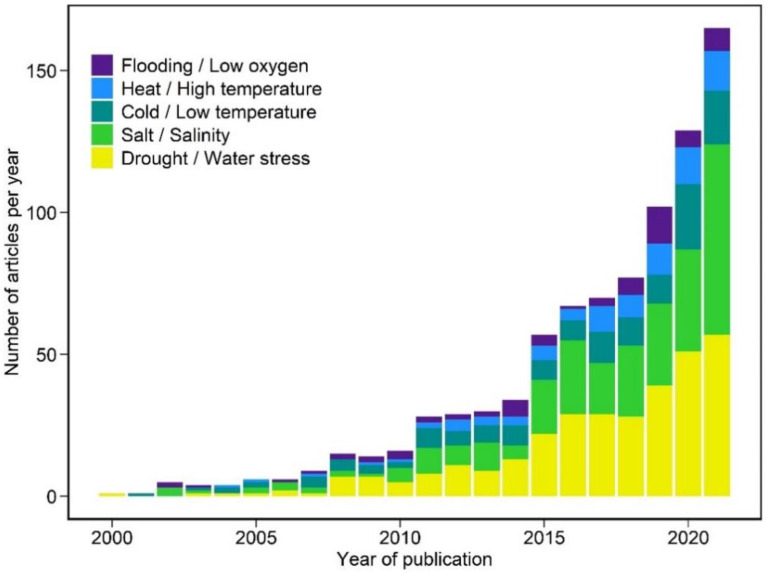
Number of publications per year from a Web of Science core collection search for research articles on metabolomics or metabolic profiling applied to plant response to major abiotic stresses from 2000 to 2021.

Metabolomics is an emerging field in the post-genomic era that enables scientists to better understand an organism’s physiological state and response to stimuli. Metabolomics is the comprehensive, quantitative, and qualitative analysis of the metabolome, the complete set of small molecules in a biological system [39]. As the final product of cellular regulatory processes, metabolites provide a more direct representation of the phenotype than genes and proteins whose functions are affected by epigenetic regulation and post-translational modifications [1]. The application of metabolomics has become increasingly common in studying plant responses to abiotic stresses. Searching for research articles in the Web of Science core collection revealed that research using metabolomics or metabolic profiling to study plant abiotic stress responses has progressively increased over the past two decades (Figure 1). There was a drastic increase in publications from 2018 to 2021, with many of them focusing on drought, salinity, and cold responses (Figure 1).

**Figure 2:**
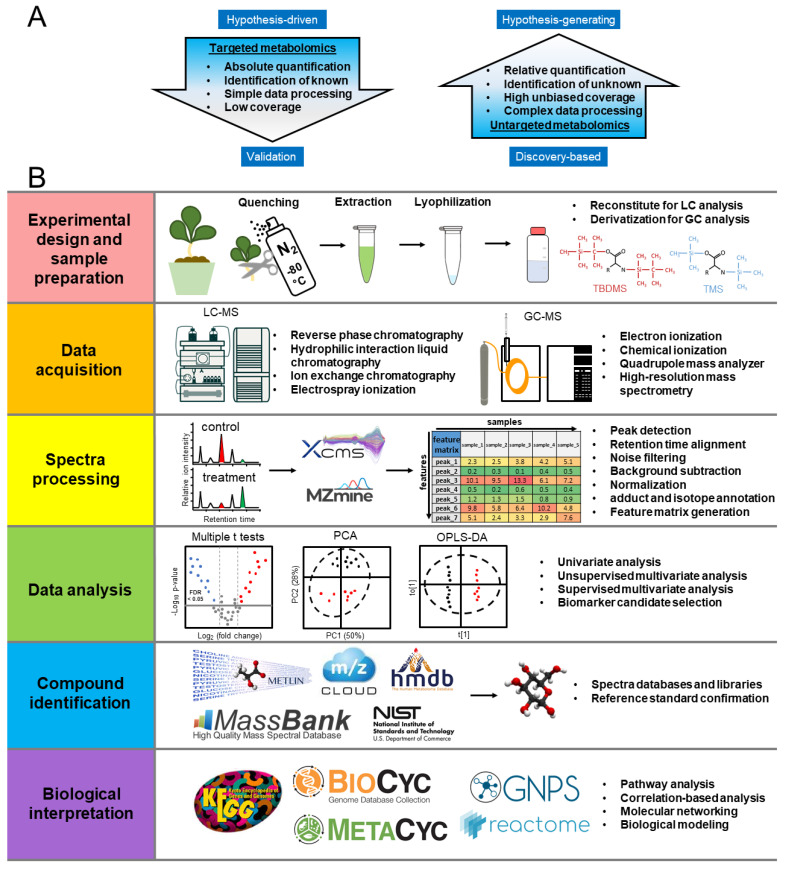
(**A**) Targeted and non-targeted metabolomics. (**B**) Protocol workflow of plant metabolomics studies. Abbreviations: GC-MS: gas chromatography-mass spectrometry; LC-MS: liquid chromatography-mass spectrometry; PCA: principal component analysis; OPLS-DA: orthogonal projections to latent structures discriminant analysis.

Metabolomic analysis can be classified as either non-targeted or targeted. The non-targeted analysis focuses on the pattern-based classification of as many metabolites as possible in the system with unbiased and global screening (Figure 2A). Non-targeted metabolomics is more commonly used for discovery-based questions, such as the characterization of the most dramatic metabolic changes, when comparing stress treatments and the control [40]. Non-targeted metabolomics has advantages of high unbiased coverage, but faces challenges in complex data-processing processes, relative quantification, and identification of unknowns. In contrast, targeted metabolomic analysis focuses on the identification, quantification, and interpretation of specific responses, and is more often employed to address questions in a hypothesis-driven manner [17,41]. Compared to non-targeted metabolomics, targeted metabolomics focuses on known metabolites with simple data processing, absolute quantification, but low coverage. Both non-targeted and targeted metabolomics analyses have been commonly used in characterizing plant responses to abiotic stresses.

Evolving metabolomics approaches provide a new opportunity to capture the metabolic changes under abiotic stresses. The most widely used analytical technologies in metabolomics research include nuclear magnetic resonance (NMR) spectroscopy and mass spectrometry (MS) [42]. The NMR-based metabolomic analysis is non-destructive and powerful in providing structural information about the metabolites [43]. Although MS-based metabolomic analysis is destructive, it has gained popularity because of its high sensitivity in metabolite detection [42]. MS approaches are often coupled with chromatographic separation techniques, such as gas chromatography (GC) and liquid chromatography (LC) [42]. The separation of the complex biological samples with a mixture of metabolites before ion detection aids in distinguishing isobaric compounds that have a similar mass. Alternatively, metabolites may be directly measured by the direct-infusion mass spectrometry (DIMS) approach without prior chromatographic separation [44]. DIMS has been extended to rapid, high-throughput fingerprinting strategies using high-resolution mass spectrometers, such as Fourier Transform Ion Cyclotron Resonance (FT-ICR) mass spectrometers [45]. However, no single analytical platform can cover the entire metabolome due to the broad range of chemical properties of metabolites and wide variation in their cellular abundances.

The metabolomics workflow incorporates six steps, as shown in Figure 2B, including experimental design and sample preparation, data acquisition, spectra preprocessing, data analysis, compound identification, and biological interpretation [46]. First, experimental design should define the biological questions to be addressed with appropriate quality controls, such as reagent blanks and sample pools [46]. After sample collection and extraction, the data are collected from different analytical instruments. The choice of instruments should be based on the chemical properties of metabolites of interest. LC-MS can analyze a wide variety of metabolites from polar to non-polar by the selection of columns, such as reversed-phase, hydrophobic interaction, or ion exchange columns [47]. The electrospray ionization (ESI) commonly utilized in LC-MS can be operated in both positive and negative modes to increase the metabolite coverage [48]. However, LC-MS generates adducts that can complicate analyses, and it has less consistent retention indices and spectra libraries than GC-MS [39]. GC-MS is suitable for volatile compounds that can be ionized by electron ionization (EI) or chemical ionization (CI) modes. Trimethylsilyl (TMS) and tert-butyldimethylsilyl (TBDMS) derivatization are suitable for the GC-MS analysis of a wide variety of central metabolites, including amino acids, organic acids, and sugars [49]. Compared to LC-MS, GC-MS has higher reproducibility with larger spectral libraries for metabolite annotation [48].

After data collection, the spectra can be preprocessed for peak detection, retention time alignment, noise filtering, background subtraction, normalization, and annotation of adducts and isotopes. After spectra processing, the feature matrix of peak relative abundance can be generated for further statistical analysis. A variety of statistical approaches can then be performed by univariate, unsupervised multivariate, and supervised multivariate analysis [50,51]. Univariate statistics, such as the *t*-test and analysis of variance (ANOVA), can test hypotheses on each metabolite of interest and measure significance, which is commonly used in targeted metabolomics [52]. In comparison, multivariate statistics, such as unsupervised multivariate techniques, including principal component analysis (PCA), and supervised multivariate techniques, such as partial least squares discriminant analysis (PLS-DA) and orthogonal projections to latent structures discriminant analysis (OPLS-DA), are widely used in untargeted metabolomics for global matrix dataset. Unsupervised multivariate techniques are particularly useful for sample clustering to show groups of observations, trends, and outliers [53]. Once the trend is identified, supervised techniques, such as OPLS-DA, can be used for greater discrimination power. The variable importance in projection (VIP) scores for OPLS-DA is commonly used for the selection of biomarker candidates [46]. After data analysis, the compounds of interest can be putatively identified by spectra databases and then confirmed by reference standards.

Finally, the biological interpretation of those confidently identified metabolites can be linked back to metabolic networks by pathway analysis, correlation-based analysis, molecular networking, and biological modeling to produce insight into their biological functions [46,54]. Metabolomics and its integration with other omics platforms has been actively developed in recent years, including pathway databases and viewers, and molecular networking tools, such as KEGG (http://www.genome.ad.jp/kegg/, accessed date: 15 April 2022) [55,56], BioCyc (http://biocyc.org/, accessed date: 15 April 2022) [57], MetaCyc (http://metacyc.org/, accessed date: 15 April 2022) [58], Reactome (https://reactome.org/, accessed date: 15 April 2022) [59], and GNPS (https://gnps.ucsd.edu/, accessed date: 15 April 2022) [54].

## 3. Plant Central Metabolism as a Hub to Respond to Abiotic Stress

### 3.1. Flooding Stress

The major damage to plants from soil flooding is oxygen deprivation, which negatively affects mitochondrial respiration [14]. When the oxidative phosphorylation of the mitochondrial respiration is impaired under anaerobic conditions, respiratory adenosine triphosphate (ATP) production drops substantially [60]. To cope with the energy crisis, plants increase the glycolytic flux to produce more ATP via a faster depletion of sugar reservoirs [14]. In such stress conditions, plants must generate sufficient ATP to maintain cellular functions and regenerate oxidized NAD^+^ to maintain the glycolytic flux. Pyruvate accumulated from glycolysis can be channeled through fermentation pathways to restore the pool of NAD^+^ required for glycolysis [60].

Ethanol fermentation and lactate fermentation are the two fermentation pathways in plants that use pyruvate as the substrate. In ethanol fermentation, pyruvate is decarboxylated to acetaldehyde via pyruvate decarboxylase (PDC) and then reduced to ethanol via alcohol dehydrogenase (ADH) with concomitant oxidation of NADH to NAD^+^ [61]. Due to the substantially lower energy yield of ethanol fermentation (2 mol ATP per mol glucose consumed), as compared to mitochondrial respiration (36–38 mol ATP per mol glucose consumed), ethanol fermentation must proceed at higher rates to meet the energy demand of cellular functions [62]. Accumulation of the volatile and phytotoxic ethanol and acetaldehyde has been measured in various tree and grass species exposed to flooding [63,64,65]. In flooding tolerant trees, a large amount of ethanol produced from ethanol fermentation in flooded roots could be transported to leaves via the transpiration stream, where it is sequentially oxidized to acetaldehyde and acetate via ADH and aldehyde dehydrogenase in leaves [65,66]. Acetate is converted into acetyl-CoA via acetate-activating enzymes and re-enters central metabolism, which recovers carbon that would otherwise be lost as ethanol in hypoxic tissues [67]. In lactate fermentation, pyruvate is reduced to lactate by lactate dehydrogenase with concomitant oxidation of NADH [68]. Because lactate is a weak acid, its accumulation could cause cellular acidification, potentially leading to the inactivation of enzymes and cell damage [69].

In addition to the adjustment in carbon metabolism via ethanol and lactate fermentation, oxygen deprivation also greatly affects nitrogen metabolism in plant cells [70]. Alanine is one of the most dramatically accumulated amino acids upon oxygen deficiency [71]. The major route for anaerobic accumulation of alanine is via alanine aminotransferase (AlaAT), which favors the conversion of pyruvate and glutamate to alanine and 2-oxoglutarate under hypoxia [72]. How do plants regenerate glutamate as the substrate for AlaAT under hypoxia? The reductive amination of 2-oxoglutarate via the NADH-dependent glutamate synthase (NADH-GOGAT) may be responsible for the newly synthesized glutamate under hypoxia [73]. The increased NADH-GOGAT activity also regenerates NAD^+^ needed for maintaining the glycolytic flux upon oxygen deficiency [70]. Another route for anaerobic accumulation of alanine is via a process known as γ-aminobutyric acid (GABA) shunt, where glutamate-derived GABA is converted to succinic semialdehyde, concomitantly converting pyruvate to alanine [74]. The accumulation of alanine and GABA has been proposed as an adaptive mechanism under hypoxia to safeguard the carbon that would be otherwise lost during ethanol fermentation and save the ATP that would be used otherwise for assimilating glutamine and asparagine via ATP-consuming enzymes [74]. Changes in many other amino acids, such as aspartate, glutamate, and tyrosine, have been observed in several species under flooding stress [75,76,77,78,79]. In addition, photorespiratory intermediates, such as serine, glycine, glycolate, and glycerate, increased in roots of *Medicago truncatula* under waterlogging, suggesting a higher photorespiration rate, probably due to the lower stomatal conductance [76].

The TCA cycle operates in noncyclic mode upon oxygen deficiency [73]. Anaerobic accumulation of alanine is accompanied by the production of 2-oxoglutarate, which can enter mitochondria to form succinate via 2-oxoglutarate dehydrogenase and succinate CoA ligase, generating additional ATP to alleviate the energy shortage due to the oxygen limitation. The mitochondrial NAD^+^ required to oxidize 2-oxoglutarate is generated by reducing oxaloacetate to malate via malate dehydrogenase [75]. Because the TCA cycle enzyme succinate dehydrogenase (SDH) requires oxygen, the accumulation of succinate is typical during hypoxia conditions induced by flooding [73]. Changes in other TCA cycle intermediates, such as citrate, malate, and fumarate, have occurred in several species under flooding stress [75,76,77,78,79].

### 3.2. Drought Stress

The low water availability in drought-stressed plants limits photosynthesis and restricts plant growth and development [80]. The decline in net CO_2_ assimilation under a water limitation is due to the decreased CO_2_ diffusion from the atmosphere to the sites of carboxylation within chloroplasts, which is caused by stomatal closure and probably also the increased mesophyll diffusional resistance [80]. The diffusional resistances of CO_2_ under water deficits are thought to restrict photosynthesis more directly than the metabolic limitations under water stress [81]. As photosynthesis is the major sink for photosynthetic electrons, water-stressed leaves with decreased photosynthesis are subjected to excess energy, leading to ROS formation that can impair ATP synthesis [82,83]. There is evidence that the activity of ribulose-1,5-bisphosphate carboxylase-oxygenase (Rubisco) decreases under water stress [84], which could be related to decreased ATP and Rubisco activase activity [82]. As CO_2_ availability is decreased, photorespiratory flux relatively increases in leaves of C_3_ plants under water deficit, contributing to electron sinks and resulting in high rates of H_2_O_2_ production [85]. The imbalance between the supply and demand of ATP or NADPH may be the main factor driving the metabolic pool-size changes induced by drought stress [86,87].

Osmotic adjustment, the accumulation of solutes, is one of the main strategies plants use to maintain positive turgor pressure in water-limited environments [88]. The osmolytes that are accumulated following drought stress are chemically diverse, including soluble sugars (e.g., glucose, fructose, sucrose, and trehalose); the raffinose family oligosaccharides (RFOs, e.g., raffinose, galactinol, and myo-inositol); amino acids (e.g., proline and GABA); quaternary ammonium compounds (e.g., glycine betaine); and polyamines (e.g., putrescine and spermidine) [27,89]. Many of these osmolytes are also involved in other abiotic stresses, such as salinity, cold, and flooding [90]. Soluble sugars are not only important for osmoregulation and the balance between the supply and utilization of carbon and energy in water-stressed plants; they also function as signaling molecules governing many changes in physiology and development [91]. Multiple time-course experiments revealed that sugars, such as RFOs, glucose, and fructose, generally accumulate earlier and more rapidly than many other metabolites in response to drought stress [92,93].

The accumulation of amino acids, such as proline and GABA, occurs later than sugars in response to drought [89,93]. Increased pools of amino acids require more nitrogen assimilation, which is inhibited when ATP is limited in the stressed plants. An alternative source of ammonium would be via glutamate dehydrogenase (GDH), which reversibly catalyzes the formation of glutamate by the amination of 2-oxoglutarate produced from the TCA cycle [94]. The GDH may become important for ammonium assimilation when plants are ATP-limited under drought stress, evidenced by the increased GDH activity in drought-stressed plants with the concomitant rise in proline levels [95]. The increase in branched-chain amino acids (BCAAs), such as leucine, isoleucine, and valine, is commonly observed in many plant species under drought stress [96,97]. The accumulation of BCAAs is probably associated with the high demand for the catabolism of BCAAs to fuel the alternative pathways of mitochondrial respiration during drought stress [97].

### 3.3. Cold Stress

Cold stress impairs plant development, reduces plant growth and development, and causes crop economic loss. Cold stress can lead to various plant symptoms, including poor germination, stunted seedlings, yellowing of leaves, reduced leaf expansion and wilting, and severe membrane damage caused by acute dehydration associated with the formation of ice crystals [18]. The molecular basis and regulatory mechanisms for plant cold stress responses have been widely studied, including Ca^2+^ fluxes, inositol phosphates, mitogen activated protein (MAP)-kinase-mediated cascades, Ca-dependent protein kinases, and many transcription factors. Inducer of CBF Expression-1 (ICE1) and the C-repeat-binding factors (CBFs) are best-characterized transcripts that control an important regulon of target genes that include many of the downstream core genes [98,99]. About 10–15% of all the cold-regulated genes are activated by transcriptional activators C-repeat-binding factors/dehydration responsive element-binding factors (CBF1/DREB1b, CBF2/DREB1c, CBF3/DREB1a) [100,101].

Cold stress regulates GABA shunt and the accumulation of proline, raffinose, and galactinol [102,103]. Cold stress-induced transcripts for genes encoding enzymes involved in the induction of callose, fermentation, phospholipid, starch, sugar, flavonoid, protein amino acids, GABA, and terpenoid biosynthesis, and the repression of photorespiration, folic acid, betaine, sulfate assimilation, ethylene, fatty acid, gluconeogenesis, amino acids, brassinosteroids, and chlorophyll biosynthesis [102]. Metabolomic responses to cold stress have been widely studied in *Arabidopsis thaliana* traditionally and have recently expanded to crop, grass, and medicinal plants [104,105,106]. Cold stress was found to cause more changes to metabolite levels than heat stress [102,103]. Cold stress leads to an increase in a diverse range of metabolites, including proline, GABA, soluble sugars (e.g., glucose, fructose, inositol, galactinol, raffinose, sucrose, and trehalose), ascorbate, putrescine, citrulline, TCA-cycle intermediates, polyamines, and lipids [103,107,108,109,110,111]. Plants under cold stress showed an increase in the proportion of unsaturated fatty acids to stabilize the membranes and maintain membrane fluidity against freeze injury [102,103,112,113,114].

### 3.4. Heat Stress

Heat stress can disrupt plant physiology by reducing membrane stability and inhibiting respiration and photosynthesis [115,116]. Heat and cold stresses shared many common responses, including the induction of osmolytes that function to reduce cellular dehydration, compatible solutes that are important to stabilize enzymes and membranes, chelating agents that can neutralize metals and inorganic ions, and energy sources [102,109,117].

Plants under heat shock and prolonged warming showed different responses. In response to heat shock, plants produce heat-shock proteins (HSPs) that function as molecular chaperons to defend against heat stress [118]. The heat-shock response is regulated by the transcription factor HSFs family. Part of heat-shock-affected genes was controlled by two major HSF genes, HsfA1a and HsfA1b [119]. HSFA1a/1b regulated genes encoding enzymes involved in signaling, transport processes, and the biosynthesis of osmolytes.

Several metabolomics studies have revealed the impacts of heat shock on plant central metabolism, including amino acids, organic acids, amines, and carbohydrates. Amino acids derived from oxaloacetate and pyruvate (asparagine, leucine, isoleucine, threonine, alanine, and valine), oxaloacetate precursors (fumarate and malate), amine-containing metabolites (β-alanine and GABA), and carbohydrates (maltose, sucrose, trehalose, galactinol, myo-inositol, raffinose, and monosaccharide cell-wall precursors) were reported to increase in response to heat shock [3,58,103,120]. The increase in free-amino acids during heat stress was associated with the breakdown of proteins [58,120]. The increase in the TCA-cycle intermediates under heat stress suggests that higher amounts of Coenzyme A may be important for increased biosynthetic and energy needs [103]. The induction of the raffinose biosynthesis pathway and accumulation of galactinol and raffinose during heat shock were mediated by galactinol synthase-1 (GolS1) controlled by HSFs [119]. In contrast to the short-term heat shock, plants exposed to prolonged warming enhance the glycolysis pathway but inhibit the TCA cycle [121]. Wheat (*Triticum aestivum*), under prolonged warming, showed an increase in tryptophan [122]. Cytokinins (CKs), fatty acid metabolism, flavonoid, terpenoid biosynthesis, and secondary metabolite biosynthesis were identified as the most important pathways involved in prolonged warming response [122].

### 3.5. Salinity Stress

Salinity stress negatively impacts plants’ water and nutrients uptake, growth and development, photosynthesis, and protein biosynthesis [123]. Salinity stress may induce both osmotic and ion stresses [124]. A previous study showed that the high-voltage electrical discharge treatment could improve the germination and early growth of wheat in drought and salinity conditions [125]. The main difference between osmotic adjustment induced by salinity and drought stresses is the total amount of water available. In addition to low water potential, the concentration of harmful ions, such as Na^+^, Cl^−^, or SO_4_^2−^, increased associated with salinity stress, causing specific ion toxicity effects [126]. NaCl is the most abundant salt in plants under salinity stress. A high concentration of Na^+^ and/or Cl^−^ in cells inhibits photosynthesis [127]. The transport systems, such as K^+^–Na^+^ transporter (HKT1), Na^+^–H^+^ antiporter SOS1 (salt overly sensitive 1) AtNHX1, and calcium-regulated transporters SOS2/SOS3, are important in regulating Na^+^ compartmentation during salinity stress [128,129,130,131,132].

Metabolomics has been extensively used to characterize the salinity responses of various plant species. Central metabolites, including sugars, polyols, and amino acids, play important roles in osmotic adjustment, cell turgor pressure maintenance, signaling molecules, carbon storage, and free-radical scavenging [17]. A variety of plants under salt stress were reported to accumulate osmolytes as soluble sugars (sucrose, trehalose, and raffinose) and sugar alcohols (sorbitol, galactinol, and mannitol) [133,134,135,136,137,138]. Amino acids, such as proline, can also function as osmolytes to protect plants under salt stress in many varieties [38,139,140,141]. For example, Tibetan wild barley (*Hordeum spontaneum*) and cultivated barley (*H. vulgare*) under salt stress were reported with changes in amino acids, including proline, alanine, aspartate, glutamate, threonine, and valine, with genotype-dependent manners [142]. Eight amino acids and amines, including 4-hydroxy-proline, asparagine, alanine, arginine, phenylalanine, citrulline, glutamine, and proline, were reported to be significantly increased in multiple barley varieties under salt stress [138]. Both *Thellungiella halophila* and *Arabidopsis thaliana* under salinity stresses showed an increase in proline and sugars. *Triticum durum Desf.* Exposed to salinity stress showed an accumulation in proline, GABA, threonine, leucine, glutamic acid, glycine, mannose, and fructose, and the depletion of organic acids, including TCA-cycle intermediates [143]. Rice (*Oryza sativa*) pretreated with chemical priming reagent hydrogen sulfide (H_2_S) showed better growth and development under salt stress with elevated levels of ascorbic acid, glutathione, redox states, and the enhanced activities of ROS- and methylglyoxal-detoxifying enzymes [17].

The biomarkers for salt-tolerant varieties vary between species. Three halophytes, *Sesuvium portulacastrum*, *Spartina maritima*, and *Salicornia brachiate*, were compared under salinity stress [144]. Proline increased in *Sesuvium portulacastrum* and *Spartina maritima*, while glycine betaine and polyols increased in *Spartina maritima* and *Salicornia brachiate* [144]. Salinity-resistant *Lotus japonicus* seedlings showed an increase in threonine, serine, ononitol, glucuronic acids, and gulonic acids, and decreased asparagine and glutamine [145]. Salt-tolerant cultivar barley (*Hordeum vulgare*) showed increased proline, carbohydrates, hexose phosphates, and TCA-cycle intermediates [142,146]. Salt-tolerant rice (*Oryza sativa*) showed increased concentrations of amino acids, serotonin, and gentisic acid, and decreased concentrations of TCA intermediates [147]. Salinity-resistant transgenic tobacco (*Nicotiana*) plants showed an increase in proline, glutathione, and trehalose, and a decrease in fructose [148]. Omeprazole-treated tomato (*Solanum lycopersicum*) with improved salinity tolerance showed increased polyamine conjugates, alkaloids, sesquiterpene lactones, and abscisic acid, and a decrease in auxins and cytokinin, and gibberellic acid [149]. Sugar-beet (*Beta vulgaris subsp. vulgaris*) seedlings under salinity stress showed an increase in malic acid and 2-oxoglutaric acid in the short-term treatment and an increase in betaine and melatonin in the long-term treatment [150]. Hulless barley (*Hordeum distichon*) under salinity stress showed increased tryptophan, glutamic acid, phenylalanine, cinnamic acid, inosine 5-monophosphate, and abscisic acid [151].

## 4. Common and Unique Metabolic Changes in Central Metabolites under Abiotic Stresses

**Figure 3:**
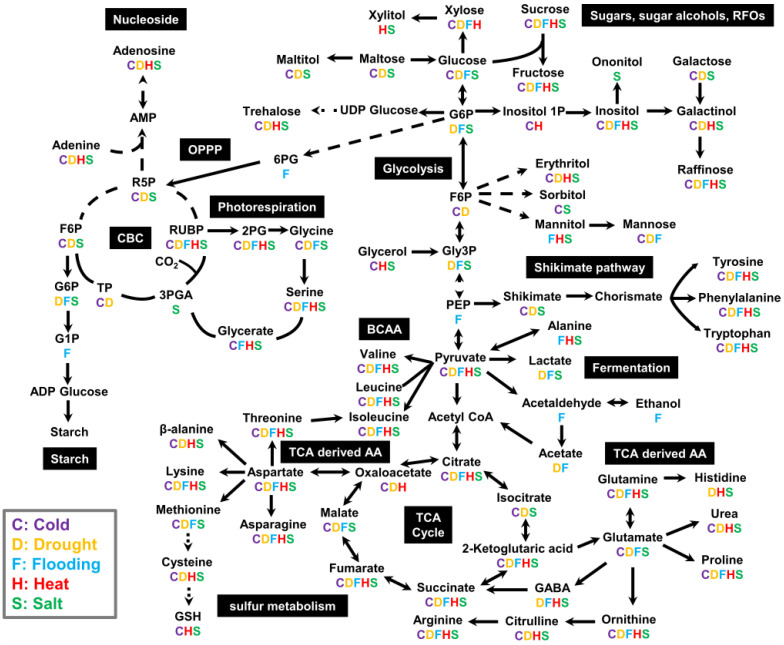
Summary scheme showing the significantly changed central metabolites under cold, drought, flooding, heat, and salinity stress. The letter below each metabolite indicates that the metabolite is significantly changed under the specific stress. Depicted data are extracted from published studies shown in Table 1. Abbreviations: 2PG: 2-phosphoglycolate; 3PGA: 3-phosphoglyceric acid; 6PG: 6-Phosphogluconate; ADP Glucose: adenosine diphosphate glucose; AMP: Adenosine monophosphate; CBC: the Calvin–Benson–Bassham cycle; F6P: fructose-6-phosphate; G1P: glucose-1-phosphate; G6P: glucose-6-phosphate; GABA: gamma-aminobutyric acid; Gly3P: Glycerol 3-phosphate; GSH: reduced glutathione; OPPP: the oxidative pentose phosphate pathway; PEP: phosphoenolpyruvate; R5P: ribose-5-phosphate; RUBP: ribulose-1,5-bisophosphate; TCA: the tricarboxylic acid cycle; UDP Glucose: uridine diphosphate glucose.

Plants exhibit diverse metabolic responses to different abiotic stresses. We reviewed the published literature for metabolomics studies on plant abiotic stress responses, including cold, heat, drought, flooding, and salinity stresses, and summarized key metabolites in major metabolic pathways in central metabolism that are affected by each stress (Figure 3, Table 1). Among the summarized metabolites, 52 were affected by cold stresses, 55 by drought, 46 by flooding, 42 by heat, and 58 by salinity. Some stress-related metabolites showed common responses to multiple abiotic stresses, and some metabolites only responded to specific stress. Interestingly, 23 metabolites showed common stress responses to all stresses reviewed in this study, including cold, heat, drought, flooding, and salinity.

As leaf photosynthesis and photorespiration are highly sensitive to environmental changes, many intermediates in the CBC and photorespiratory pathway respond to various abiotic stresses (Figure 3). When the absorbed light energy exceeds its consumption, oxidative damage occurs and causes alterations in plant central metabolism under most abiotic stresses. The common trends for metabolic changes in response to oxidative damage include the prevention of ROS formation, maintenance of essential metabolite biosynthesis, and accumulation of protective compounds, such as compatible solutes or osmolytes to protect plants from oxidative damage [195,196]. Various abiotic stresses trigger the accumulation of compatible solutes, such as sucrose, trehalose, raffinose, mannitol, sorbitol, inositol, and proline (Figure 3). These molecules have properties of high solubility in water, non-toxicity at high concentrations, and reduction in protein–solvent interactions at low water activities [109,197]. Introducing biosynthetic pathways for these compatible solutes can increase crop abiotic stress tolerance, which has been of interest to metabolic engineering [197,198].

The shikimate pathway is activated under various abiotic stresses, which leads to the accumulation of aromatic amino acids, such as tyrosine, phenylalanine, and tryptophan (Figure 3). These aromatic amino acids are the precursors for the biosynthesis of flavonoids, alkaloids, and phytoalexins with antioxidant properties [199]. Sulfur-containing amino acids and sulfur-containing metabolites, such as methionine, cysteine, and glutathione, are also induced by various abiotic stresses (Figure 3). These metabolites have important roles in plant antioxidant systems [200,201,202]. The synthesis of cysteine and its product glutathione are modulated by the ratio between reduced and oxidized glutathione [203,204].

Oxidative damage induced by various abiotic stresses can lead to the redistribution of glycolytic carbon flux into the oxidative pentose phosphate pathway (OPPP) and perturbation of the TCA cycle, resulting in the accumulation of sugar phosphates related to glycolysis and OPPP and decreases in TCA-cycle intermediates and TCA-cycle-derived amino acids [205,206]. Common stress responses have been observed in glutamate (derived from 2-oxoglutarate) and aspartate (derived from oxaloacetate), both of which are important substrates for other TCA-cycle-derived amino acids. In addition, the nonessential amino acid citrulline (derived from glutamate) also functions as an antioxidant and efficient hydroxyl radical scavenger [207,208].

GABA is rapidly accumulated under various abiotic stresses [209,210,211]. The main function of GABA includes being a signaling molecule, osmoregulator, and regulation of cytosolic pH [209]. BCAAs (valine, leucine, and isoleucine) and other amino acids sharing synthetic pathways with BCAA (lysine, threonine, and methionine) generally have similar patterns with GABA that are accumulated in response to various abiotic stresses [178,212]. The accumulation of BCAAs are either from the activation of the biosynthetic pathway or protein degradation [178,212]. BCAAs are critical for normal plant growth and can function as compatible solutes, alternative electron donors for the mitochondrial electron transport chain, and substrates for protective secondary metabolites, such as cyanogenic glycosides, glucosinolates, and acyl-sugars [178,212,213].

Changes in ethanol fermentation intermediates, such as ethanol, acetaldehyde, and acetate, are more specific to the flooding stress (Figure 3). The ability to recover the carbon that would be lost as ethanol by converting it into acetate and then acetyl-CoA to support the flooded plant’s carbon metabolism could be related to plant flooding tolerance [66]. Acetate is also recently identified as a key metabolite that mediates a novel drought-survival mechanism [189]. The metabolic switch from glycolysis to acetate biosynthesis via pyruvate decarboxylase and aldehyde dehydrogenase stimulates the jasmonate signaling pathway for plant drought tolerance [189]. Furthermore, engineering increased the expression of the acetate biosynthesis pathway, and the exogenous application of acetic acid enhanced plant survival under drought stress [24,214,215].

## 5. Challenges and Perspectives in Deciphering Plant Metabolic Responses to Abiotic Stresses in Time and Space

A major challenge in characterizing plant metabolism under stress is the compartmentalization at the level of cells and organelles. Distinct tissue-specific metabolic responses to abiotic stresses have been characterized in many plant species [76,216,217]. In contrast, our knowledge of cell-specific and organelle-specific metabolic responses has been hampered by the limitations of current analytical methods to determine the cellular and subcellular location of metabolites. Although aqueous fractionation methods have been used to analyze cell-specific and organelle-specific metabolites [218,219], it is laborious and requires extensive optimizations to reduce the risk of contamination, low coverage, and missing compartments [220]. The other challenge in capturing the plant metabolome during stresses is the quenching step that stops the metabolism faster than the turnover of metabolites to preserve the metabolic profile at the point of the stress treatment applied. Because turnover times of many metabolites are in the order of seconds or less [221], it is essential to ensure the fast harvest and quenching of samples, which can be difficult to apply in field conditions.

Another major challenge in characterizing plant metabolism under stress is the identification of unknown metabolites due to complex and dynamic plant metabolism, comprehensive detection methods in databases, instruments, and platforms. The plant kingdom is estimated to contain between 200,000 and 1,000,000 metabolites [21]. Due to this great chemical diversity and the wide range in concentrations, to date, there is no single instrument platform for the comprehensive examination of the whole metabolome [222]. Recently, liquid chromatography-high resolution-mass spectrometry (LC-HR-MS) has become a leading technology for metabolomics because it provides a more universal view of the metabolism [1,47]. However, it is accompanied by major challenges in metabolite annotation [223].

The identification of unknowns by LC-MS is complicated and challenging because of no uniform databases and platforms. The three-level identification includes MS1 spectral identification, MS2 spectral identification, and authentic standard identification. First, putative identification can be performed by query of MS1 compounds and spectral databases, such as METLIN (https://metlin.scripps.edu, accessed date 15 April 2022) [224], ChemSpider (http://www.chemspider.com, accessed date 15 April 2022), Human Metabolome DataBase (HMDB) (https://hmdb.ca, accessed date 15 April 2022), KEGG (https://www.genome.jp/kegg, accessed date 15 April 2022) [55], MassBank (https://massbank.eu/MassBank, accessed date 15 April 2022) [225], mzCloud (https://www.mzcloud.org, accessed date 15 April 2022), and GNPS (https://gnps.ucsd.edu, accessed date 15 April 2022) [54]. However, even the high-mass accuracy measurement with less than 1 ppm error is not sufficient to always fully determine the elemental composition [226]. After putative identification by MS1, further information, such as MS2 fragmentation data, is needed to perform the MS2 spectral database search. Nonetheless, the spectra databases for MS2 searching are currently considered incomplete because the fragmentation patterns are greatly affected by instrument, collision energy, and ionization source. The scores for MS2 searching can often result in false positive and false negative results [227]. Therefore, to confidently identify the metabolite, authentic standards are always needed [46].

While metabolomic profiling has become increasingly common in plant-stress physiology studies, the measurement of metabolite abundances alone is insufficient to inform the activity of the metabolic pathways involved and the time-dependent flux changes in response to abiotic stresses. Stable isotope-assisted metabolomics enables global assessment of fluxes through plant metabolic networks [228]. Typically, an isotope-labeled precursor is introduced into plant tissues, and the redistribution of the label from the precursor Into downstream metabolites is detected by MS or NMR [229]. The labeling data can be coupled to mathematical modeling to construct flux maps, representing a quantitative description of metabolic phenotypes [230]. Transient labeling-based flux approaches, such as isotopically nonstationary metabolic flux analysis (INST-MFA), have been applied in several plant species to examine intracellular fluxes through central carbon metabolism [188,231,232]. Future applications of metabolic flux analysis in the context of abiotic stresses hold the key to elucidating the temporal dynamics of the central metabolic network under stress conditions.

Emerging advances in mass spectrometry imaging (MSI) offer a unique capability to simultaneously capture the spatial distributions of metabolites and macromolecules at the molecular level. MSI has been successfully applied to map the spatial localization of various plant metabolites, providing a mechanistic understanding of plant metabolism [233]. The most widely used MSI technique is matrix-assisted laser desorption ionization (MALDI) [234]. MALDI operates under a vacuum and has been demonstrated to visualize many lipids at a high spatial resolution of ~5 µm in maize leaves and roots [235,236]. MALDI-MSI was also used to reveal the detailed spatial distribution of lipids in barley roots in response to salinity stress [237]. In addition to MALDI, ambient ionization technique, such as electrospray laser desorption ionization, was used to study the spatial distribution of carbohydrates, organic acids, amino acids, and flavonoids in Coleus leaves in response to the change of illumination [238]. In combination with stable isotope labeling, MSI has great potential to map spatially resolved metabolic fluxes, which has been applied to mammalian tissues [239]. With improved spatial resolution and sensitivity to quantify the isotopic labeling of less abundant metabolites, stable isotope-assisted MSI holds the promise of unraveling spatially resolved metabolic flux reprogramming under abiotic stresses.

## Figures and Tables

**Table 1:** List of significantly changed central metabolites under cold, drought, flooding, heat, and salinity stress from published studies.

Classes	Metabolites	Cold	Drought	Flooding	Heat	Salinity
Amino acids	Alanine			[75,76,77,78,79]	[152]	[136,140,141,153,154,155,156,157]
	Arginine	[102,103,108,158]	[159,160,161]	[76]	[103,108]	[135,136,141,162]
	Asparagine	[103,104,105,107,108,158]	[163,164,165]	[76]	[103,108,152]	[141,142,154,155,157,166,167,168]
	Aspartate	[102,104,169]	[152,161,165,170,171,172]	[75,76]	[104,152]	[140,146,148,154,155,156,166,173]
	beta-Alanine	[158]	[159,163,164,170]	[75,76]	[103]	[142,146,168,173]
	Citrulline	[103,104,107,108,158]			[152]	
	gamma-Aminobutyrate		[152,159,165,170,171,172]	[75,76,77,79,174,175]	[103,104,152]	[135,146,154,167,176]
	Glutamate	[102,152,169]	[152,159,161,163,171,172]	[75,76,77]		[135,140,148,154,156,166,168,173,176,177,178]
	Glutamine	[102,105,107,108,158,169]	[161,164,165,179]	[75,76,78]	[146]	[140,141,155,168,173,176]
	Glycine	[152,180]	[161,163,170,171,172,179]	[75,76,77,175]	[102,103]	[135,136,140,141,142,148,154,156,162,166,176,177,181]
	Histidine		[96]		[152,178]	[135,141,178]
	Isoleucine	[102,103,104,105,158,169]	[93,96,97,161,163,164,170,171]	[78]	[104,182]	[135,140,142,155,162,168,176,177,183]
	Leucine	[102,105]	[93,152,164,170,172]	[78]	[152]	[135,136,140,142,145,162,167,177,183]
	Lysine	[102,158,169]	[93,97,164,170]	[76]	[152,182]	[135,141,145,156,178]
	Ornithine	[102,103,107,108,158]	[159,160,161]	[76]	[103,108,152]	[167,178]
	Phenylalanine	[102,105,152,158]	[93,96,97,152,161,163,164,170,171,172]	[76]	[152,182]	[136,141,146,155,156,167,181,183]
	Proline	[102,103,104,105,107,108,110,158,169]	[96,159,161,163,164,165,170,171,179]	[75]	[103,104,152,182]	[135,136,141,142,146,148,155,156,157,162,166,167,168,173,176,177,181,183,184,185]
	Serine	[102,152,158,169]	[161,163,171,172,179]	[75,76,78]	[152,182]	[141,142,146,148,156,157,162,166,167,176,181,184,186,187]
	Threonine	[102,152,158,169]	[93,97,161,163,170,172,179]	[75,78]	[104,187,188]	[135,136,140,145,146,148,168,176,187]
	Tryptophan	[103,107,108,152,169]	[93,97,164,170]	[76]	[103,108]	[135,136,147,155,183]
	Tyrosine	[102,104,105,169]	[93,97,152,163,164,170,172,187]	[75,76,78]	[104,152]	[141,147,155,156,162,167,183,187]
	Valine	[103,104,105,158]	[93,97,152,161,163,164,170,171]	[75,78]	[104,152]	[135,136,140,141,142,155,157,162,167,176,184]
CBC	3-phosphoglycerate					[142,146]
	Dihydroxyacetone phosphate	[152]	[152]			
	Fructose-6-phosphate	[103]	[152,161]			[142]
	Glucose-6-phosphate		[152]	[76,174]		[141,142,147]
	Ribose 5-phosphate	[152]	[152]			[141]
Fermentation	Acetaldehyde			[63,64]		
	Acetate		[189]	[64,190]		
	Ethanol			[63,64,65,191]		
	Lactate		[192]	[75,77]		[192]
Photorespiration	Glycerate	[103]		[78]	[103]	[168,176,192]
	Glycolate			[76]		
Glycolysis	Glycerol	[103]			[103]	[142,148,192]
	Glycerol-3-phosphate		[164]	[76]		[168]
	Phosphoenol pyruvate			[77,175]		
	Pyruvate	[152,158,169]	[96,152,170]	[77,174]	[182]	[142,147,183]
	Glucose-1-phosphate			[174]		
Nucleoside	Adenine	[105]	[96]		[152]	[185]
	Adenosine	[105]	[170]		[152]	[185]
OPPP	6-Phosphogluconate			[174]		
Shikimate pathway	Shikimate	[102,105]	[172]			[146,147,183]
Sugars	Fructose	[103,104,105,107,108,110,158,169]	[93,163,170,179,187]	[75,76]	[103,104,108,182]	[135,140,146,148,155,166,167,168,173,176,181,183,184]
	Galactose	[110,169]	[93,97,164]			[146,168]
	Glucose	[105,107,158,169]	[93,97,161,170,187,193]	[75,76]		[140,145,146,154,155,166,167,173,176]
	Maltose	[152,158]	[93,152,161,163,170]			[142,145,148]
	Mannose	[194]	[97,164,187]	[76]		
	Raffinose	[105,108,110,158,169]	[93,97,163,170,172,187,193]	[76,79]	[108,152,187]	[142,147,148,184,187]
	Ribose		[164]			
	Sucrose	[104,108,152]	[93,97,152,163,164,165,170,171,172,193]	[190]	[104,108,152]	[135,140,142,148,153,154,155,168,173,176,181,183]
	Trehalose	[103,107,158]	[97,163]		[103]	[142,146,147,148,167,168,184]
	Xylose	[103,110]	[96,164,170]	[76,79]	[103]	
Sugar alcohols	Erythritol	[103]	[93,97,161]		[103]	[135,168]
	Galactinol	[103,107,108,110,169]	[93,97,164,170]		[103,108]	[146,148,168,184,187]
	Myoinositol	[104,107,108]	[93,97,161,164,165,170,171,172,179]	[79]	[104,108,182]	[135,136,142,148,155,173,176,181,184]
	Maltitol	[110]	[164,179]			[153]
	Mannitol			[76]	[152,182]	[142,184,194]
	Myoinositol-P	[103]			[103]	
	Ononitol					[181]
	Sorbitol	[103]				[185]
	Xylitol				[182]	[142,168]
TCA	2-Ketoglutarate	[103]	[97,152,161,165]	[76,77]	[103,152]	[135,140,141,142,154]
	Citrate	[103,152,169]	[97,165,170,171]	[77,78,79,174]	[103]	[135,141,142,145,148,154,166,168,173,176,181]
	Fumarate	[103,105,110]	[93,97,161,163,164,165,170,171,172]	[76,77]	[103,182]	[135,140,141,142,148,166,168,181,183,184]
	Isocitrate	[103]	[93,97,164,171]			[135,142,146,166]
	Malate	[105,107,169]	[93,97,161,163,165,170,171,172,179]	[75,77]		[135,136,140,142,145,148,154,155,157,166,168,173,181,183,184]
	Oxaloacetate	[152]	[152]		[152]	
	Succinate	[103,110,169]	[93,96,97,164,165,170,171]	[75,76]	[103,152,182]	[140,141,142,145,148,154,157,166,167,168,181,183,184]
Sulfur metabolism	Cysteine	[102,152]	[152,171]		[103,152]	[135,141,142,148,154,156,162,166,167,181,183,184]
	GSH	[152]			[152]	[153,167,183]
	Methionine	[102,158]	[93,96,97,161,164,170,171,172]	[76]		[135,162,167]
Others	Urea	[152]	[152]		[152]	[168]

## Data Availability

No new data were created or analyzed in this study. Data sharing is not applicable to this article.
